# Prevalence of Brucellosis among Women Presenting with Abortion/Stillbirth in Huye, Rwanda

**DOI:** 10.1155/2014/740479

**Published:** 2014-06-29

**Authors:** Nadine Rujeni, Léonidas Mbanzamihigo

**Affiliations:** ^1^College of Medicine and Health Sciences, University of Rwanda, Huye Campus, P.O. Box 56, Butare, Rwanda; ^2^Veterinarians without Borders-Belgium, P.O. Box 35, Butare, Huye, Rwanda; ^3^UCL, Louvain Cooperation au Développement, Avenue Mugamba 35, P.O. Box 2076, Rohero II, Bujumbura, Burundi

## Abstract

The incidence of human brucellosis is not documented in Rwanda despite several reports on the disease in cattle. Because brucellosis has been associated with abortion, the aim of this study was to investigate the prevalence of positive serology in women presenting with abortion and/or stillbirth. The study was done in Huye District, in the Southern Province of Rwanda, and the patients were recruited from both the University Teaching Hospital of Butare (CHUB) and Kabutare District Hospital. Serum samples were collected and the Rose Bengal plate test (RBPT) was performed on each sample. A questionnaire was also used to investigate potential contacts with animals and/or consumption of raw milk. A total of 60 women were recruited and 15 (i.e., 25%) were *Brucella* seropositive. The questionnaire showed that those with seropositivity either were in contact with domestic animals (cattle, goat, or sheep) or were consuming raw cow's milk. Human brucellosis appears to be of public health importance in Rwanda and more attention should be drawn on the disease. The current study provides a basis for larger studies to establish the incidence of human brucellosis in Rwanda. More mechanistic studies will also demonstrate the pathogenicity of *Brucella* in human placentas.

## 1. Introduction

Brucellosis is a debilitating zoonotic disease due to bacteria of the genus* Brucella*. Infection is acquired either by contact with infected animals or consumption of contaminated milk or dairy products. The disease has major economic impact not only due to time lost by patients from normal daily activities but also due to the loss in animal husbandry [1, 2]. Indeed, brucellosis is characterised by abortion and loss of fertility in farm animals [2–4]. In humans, the symptomatology is not specific, and this makes the diagnostic quite challenging. Patients may present with an intermittent fever, joint pain (including arthritis), neurologic manifestations, and so forth [5]. The association between abortion/stillbirth and brucellosis in humans is controversial [6]. This could be due to the absence of erythritol (a 4-carbon sugar alcohol which is the preferred carbon source for* Brucella*) in human placentas as opposed to ruminant placentas [7, 8]. However, a recent study has demonstrated that* Brucella* replicates in several human trophoblast subpopulations and can interfere with the invasive capacity of extravillous trophoblast-like cells* in vitro* [9]. Studies investigating an association between human brucellosis and abortion* in vivo* are scarce.

In Rwanda, the incidence of human brucellosis is not documented despite a number of reports on cattle brucellosis [10]. Because of the potential risk of* Brucella*-associated abortion in infected women, the aim of this study was to establish the seroprevalence of brucellosis in women presenting with abortion/stillbirth. This study also aims to document the risk factors for human brucellosis and the potential reservoir of pathogens.

## 2. Materials and Methods

### 2.1. Study Area and Population

The study was conducted in Huye District, located in the Southern Province of Rwanda. Blood samples were collected from women presenting with abortion/stillbirth of unknown cause at the district hospital (Kabutare) and the referral hospital (Butare University Teaching Hospital).

### 2.2. Ethical Statement

Permission to conduct the study was obtained from the medical director of each hospital as well as the head of department of the gynaecology and obstetrics. Institutional approval was obtained from the National University of Rwanda (currently part of the University of Rwanda). All the prospective study participants had the aims and procedures explained to them and only those who consented were enrolled into the study.

### 2.3. Sample Collection and Processing

Blood samples were collected into silicon-coated tubes without anticoagulant for isolation of sera. The blood was stored at 4°C overnight and centrifuged the following day and the serum was extracted. The Rose Bengal plate test (RBPT) was used to determine the seropositivity. Briefly, a drop of the Rose Bengal coloured* Brucella* antigen (TRANSAK, Belgium) was placed near a drop of serum on a clean slide placed on a white paper. The drops were then thoroughly mixed and observed after 4 minutes. If an agglutination reaction occurred, the sample was recorded as a positive case (and negative otherwise).

The samples were collected from June to October 2006 and were processed all along. A total of 60 samples were collected and processed.

### 2.4. Questionnaires

A questionnaire was used to record demographic data (age, residency), clinical presentation (abortion, stillbirth), profession, and milk consumption habits.

### 2.5. Cattle Brucellosis

In order to determine the local prevalence of cattle brucellosis, records of brucellosis test results from 2 different farms were consulted. The first was the Kabutare farm, located within a veterinary school near the district hospital (Kabutare Hospital). The second was ISAR-Songa farm, part of a veterinary research institution in the Southern Province of Rwanda (ISAR-Songa is currently part of the Rwanda Agricultural Board).

Randomly selected cows (from the local population) presenting at the Kabutare farm's abattoir were also tested for brucellosis during the study period. Blood samples (27 in total) were collected from the jugular vein and serum was extracted and processed as described in the previous paragraph (RBPT).

## 3. Results

### 3.1. Brucellosis Prevalence in Women

A total of sixty women were tested for brucellosis and 15 (25%) were positive as shown in Table 1. Eleven (11) of them (73.3%) had aborted while 4 (26.7%) presented with a stillbirth. The majority (76.7%) of tested women were between 18 and 33 years old.

Table 2 shows that 13 out of the 15 seropositive women (86.7%) consumed cow's milk, with 7 of them consuming it raw. Six of the 15 women (40%) were in regular contact with farm animals by profession (livestock-farming). Two of those in contact with livestock also consumed raw milk.

### 3.2. Prevalence in Cattle from Local Farms

Records from the Kabutare veterinary school's farm showed that the prevalence of brucellosis in cattle declined (9.6% to 4.6%) from the year 2002 to 2006 (see Table 3). These records were of tests done on cattle from the local population coming for insemination (natural or artificial).

Twenty-seven (27) cows randomly selected from the Kabutare farm's abattoir were tested during the study period, and 2 cases (7.4%) were positive.

Records from ISAR-Songa farm revealed that a single test was done on their livestock in 2004 and 50 cows out of 603 (8.3%) tested positive. According to the staff in charge of the livestock, these positive cases were isolated and progressively eliminated.

## 4. Discussion

The current study, conducted in Rwanda, revealed a high prevalence (25%) of human brucellosis among women presenting with either abortion or stillbirth. To our knowledge, this is the first study to document the presence of human brucellosis in Rwanda. Moreover, the study has shown that some cases of abortion/stillbirth may be due to this pathology in humans. A recent study has demonstrated that pathogenic* Brucellae* are able not only to proliferate in human trophoblasts but also to interfere with the invasive capacity of extravillous trophoblasts-like cells* in vitro* [9].

Findings from the current study, together with findings by Salcedo and colleagues, give insights into the otherwise controversial* Brucella*-associated abortion in humans [6]. The next step will be to isolate* Brucella* spp. from placental trophoblasts of aborting women.

Results from the current study indicate that consumption of raw (cow's) milk, followed by contact with farm animals, is the main risk factor associated with* Brucella* seropositivity. This is consistent with findings from a study conducted in Iran, where 79% of infected patients contracted the disease via consumption of unpasteurized dairy products while 21% had a history of animal contact [11].

However, a lower prevalence of brucellosis in cattle (7.4%), with a declining trend in the local main farms, was observed in the current study. These findings suggest that milk from a single contaminated cow is consumed by more than one family. Moreover, while the tested cows were randomly selected, our study population was selected based on a suspicious symptomatology. The declining trend in cattle brucellosis from the consulted farms may be the result of control efforts in these institutions following documentations on cattle brucellosis [10]. There was no direct association between infected cattle and affected women in the current study as it was impossible to trace these cows specifically. However, the incidence in local farms is an indication on the local reservoir given the herds' movements and the trading of milk and dairy products.

In conclusion, this study has shown that human brucellosis exists in Rwanda and that some cases of abortion/stillbirth may be associated with this pathology. Consumption of raw milk is the principal risk factor for contamination, followed by contact with farm animals. Education of the population regarding milk consumption habits could therefore extensively reduce the prevalence of infection. This study provides a basis for a larger study to document the national prevalence of human brucellosis (for control purposes) and calls for more mechanistic studies to link brucellosis and reproductive problems in humans.

## Figures and Tables

**Table 1:** Prevalence of *Brucella* seropositivity by age group and clinical presentation.

Age range	Total number of cases		Seropositive cases		Prevalence (%)
	Clinical signs				
	Abortion	Stillbirth	Abortion	Stillbirth	
18–25	15	3	4	1	27.8
26–33	21	7	6	3	32
34–41	8	2	1	0	10
42–49	3	1	0	0	0
Total	**47**	**13**	**11**	**4**	**25**

The number of *Brucella* seropositive cases is shown for each age group.

**Table 2:** Demographic characteristics of *Brucella* seropositive patients.

Characteristic	*n* (%)
Age range	18–49 years (median = 27)
Milk consumption	13 (86.7)
Occupation	
Livestock-farming	6 (40)
Farming	6 (40)
Other	3 (20)

The age range of the study population and their occupation as well as the milk consumption rate are shown.

**Table 3:** Brucellosis prevalence in cattle for a period of 5 years in the Kabutare veterinary school's farm.

Year	Positive cases		Total cases tested	Prevalence (%)
	RBPT	Ring test		
2002	77	48	1297	9.6
2003	106	103	2290	9.1
2004	154	83	6234	3.8
2005	170	107	6925	4
2006	54	54	2340	4.6

Results from brucellosis tests (RBPT or Ring test) routinely done before any natural or artificial insemination at the Kabutare veterinary school's farm (one of the main farms of the study area). RBPT: Rose Bengal plate test.
